# Priming of mesenchymal stem cells with a hydrosoluble form of curcumin allows keeping their mesenchymal properties for cell‐based therapy development

**DOI:** 10.1111/jcmm.16403

**Published:** 2021-03-26

**Authors:** Margaux Colin, Lola Dechêne, Justine Ceusters, Ariane Niesten, Catherine Demazy, Laurence Lagneaux, Karim Zouaoui Boudjeltia, Thierry Franck, Pierre Van Antwerpen, Patricia Renard, Véronique Mathieu, Didier Serteyn

**Affiliations:** ^1^ Department of Pharmacotherapy and Pharmaceuticals Faculty of Pharmacy Université libre de Bruxelles (ULB) Brussels Belgium; ^2^ RD3‐ Pharmacognosy, Bioanalysis and Drug Discovery Unit and Analytical Platform Faculty of Pharmacy Université libre de Bruxelles (ULB) Brussels Belgium; ^3^ Department of Clinical Sciences, Anaesthesiology and Equine Surgery Faculty of Veterinary Medicine, B41 University of Liege Sart Tilman Liège Belgium; ^4^ Unité de Recherche en Biologie Cellulaire (URBC) ‐ Namur Research Institute for Life Sciences (Narilis) University of Namur (UNamur) Namur Belgium; ^5^ Centre of Oxygen, Research and Development Institute of Chemistry B6a University of Liege (ULiège) Sart Tilman Liège Belgium; ^6^ Laboratory of Clinical Cell Therapy ULB‐Research Cancer Center (U‐CRC) Jules Bordet Institute Université libre de Bruxelles (ULB) Brussels Belgium; ^7^ Laboratory of Experimental Medicine Université libre de Bruxelles CHU de Charleroi Hôpital Vésale Montigny‐le‐Tilleul Belgium

**Keywords:** curcumin, cyclodextrin, equine MSCs, mdMSC, mesenchymal stem cells, mitochondria, NDS27

## Abstract

Mesenchymal stem cells are increasingly studied for their use as drug‐carrier in addition to their intrinsic potential for regenerative medicine. They could be used to transport molecules with a poor bioavailability such as curcumin in order to improve their clinical usage. This natural polyphenol, well‐known for its antioxidant and anti‐inflammatory properties, has a poor solubility that limits its clinical potential. For this purpose, the use of NDS27, a curcumin salt complexed with hydroxypropyl‐beta‐cyclodextrin (HPβCD), displaying an increased solubility in aqueous solution, is preferred. This study aims to evaluate the uptake of NDS27 into skeletal muscle‐derived mesenchymal stem cells (mdMSCs) and the effects of such uptake onto their mesenchymal properties. It appeared that the uptake of NDS27 into mdMSCs is concentration‐dependent and not time‐dependent. The use of a concentration of 7 µmol/L which does not affect the viability and proliferation also allows preservation of their adhesion, invasion and T cell immunomodulatory abilities.

## 1 INTRODUCTION

Mesenchymal stem cells (MSCs) are multipotent stem cells implicated in homeostasis and tissue repair.^1^ Curcumin, a polyphenol extracted from the roots of *Curcuma Longa* L., is used to treat notably inflammatory diseases for thousands of years.^2^, ^3^ The major disadvantages of curcumin are its very poor water solubility and its low systemic bioavailability.^3^ The present study is therefore focused on a hydroxypropyl‐beta‐cyclodextrin (HPβCD) complex of curcumin lysinate called NDS27, which is about 33 000 times more soluble in water than synthetic curcumin.^4^ The association of MSCs with a complex of curcumin that does not require any solvent to be dissolved, may lead to a new therapeutic tool suitable for clinical application. Thus, this study aims to evaluate (a) the cellular toxicity of the NDS27 on MSCs, (b) the uptake and subcellular localization of NDS27 and (c) the effects of such loading (ie uptake) on mitochondrial function and mesenchymal properties of skeletal muscle‐derived MSCs (mdMSCs).

## 2 MATERIALS AND METHODS

Detailed materials and methods are provided in the Appendix S1 section.

## 3 RESULTS AND DISCUSSION

This *in vitro* study was specifically designed to evaluate the impact of NDS27 loading on equine mdMSCs in the aim to develop curcumin‐loaded mdMSCs. This new therapeutic association would allow possibly benefiting from both mdMSCs regenerative therapy and curcumin effects to treat inflammatory diseases.

**FIGURE 1:**
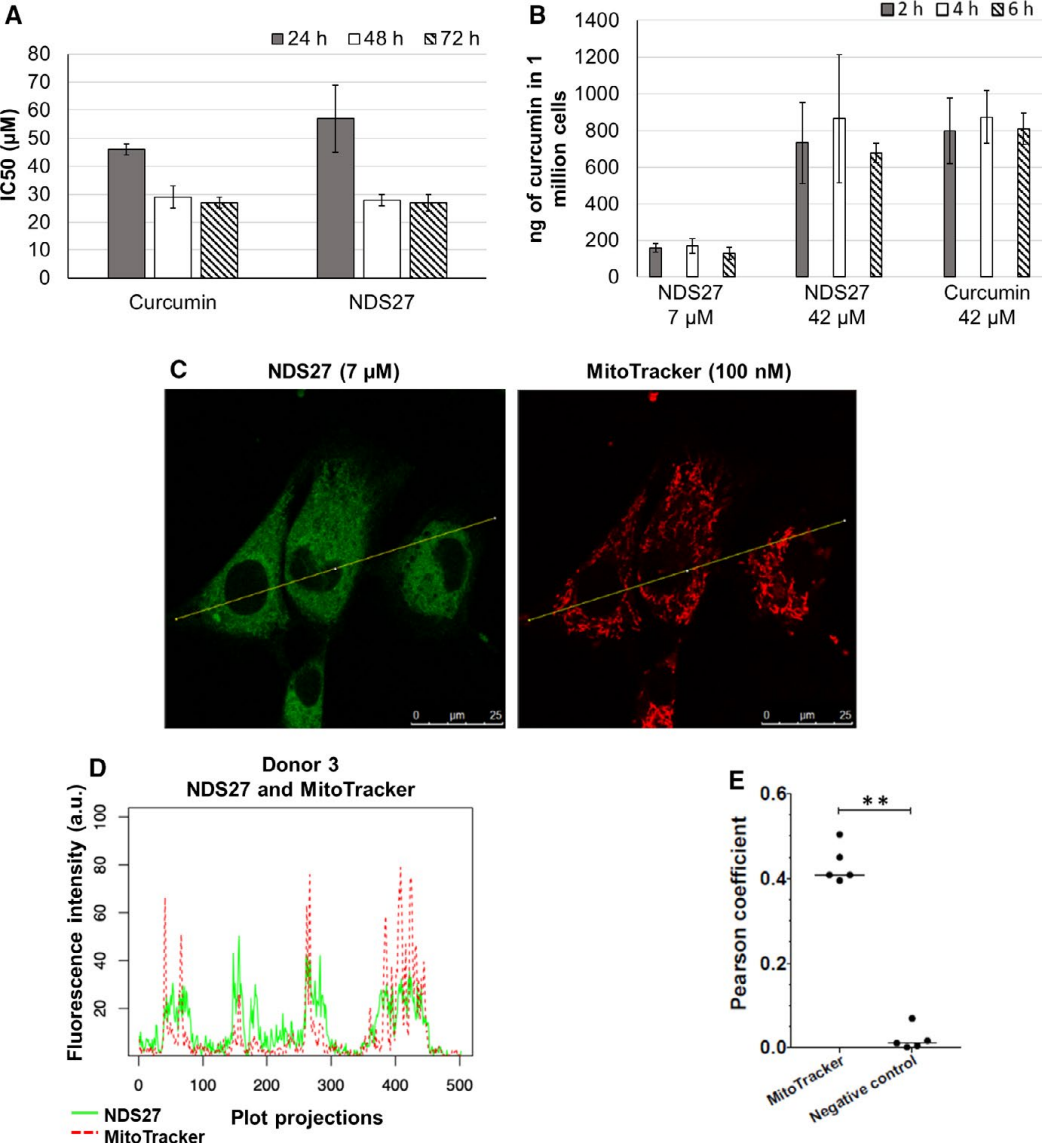
Internalization of NDS27 and synthetic curcumin by equine mdMSCs. (A) Average IC_50_ of synthetic curcumin and NDS27 on equine mdMSC after 24, 48 or 72 h of treatment relative to untreated control. Data are expressed as mean ± SD of five independent biological replicates. (B) Quantity of curcumin (ng) internalized in one million equine mdMSCs after 2, 4 or 6 h of treatment with NDS27 (7 or 42 µmol/L) or synthetic curcumin (42 µmol/L). Data are expressed as mean of three independent biological replicates ± SD. (C) Micrographies of mdMSCs from donor 3 after their loading with NDS27 at 7 µmol/L (green fluorescence; Ex: 488 nm; Em: between 502‐576 nm) and staining with MitoTracker probe (red fluorescence; Ex: 561 nm; Em: between 572‐636 nm) (magnification ×400). The thin yellow lines present on the representative micrography is the transect used for the co‐location evaluation. (D) Intensities of both green (NDS27) and red (MitoTracker) fluorescence for each point along the transect traced on the micrography shown in (C), indicating that NDS27 is partially co‐located with MitoTracker and thus, mitochondria. The presented result was obtained on donor 3 and is representative of experiments conducted on two other donors. (E) Data from 5 different donors (at least 6 images/donor) were quantified to assess the correlation of co‐location between natural fluorescence of NDS27 and MitoTracker probe using Pearson coefficient method. The correlation between NDS27 and MitoTracker is significantly distinct from the correlation between NDS27 and control condition (consisting in micrography of MitoTracker probe rotated of 90°) suggesting that the mitochondrial co‐location of NDS27 is not random. *P* <.01 (**)

**FIGURE 2:**
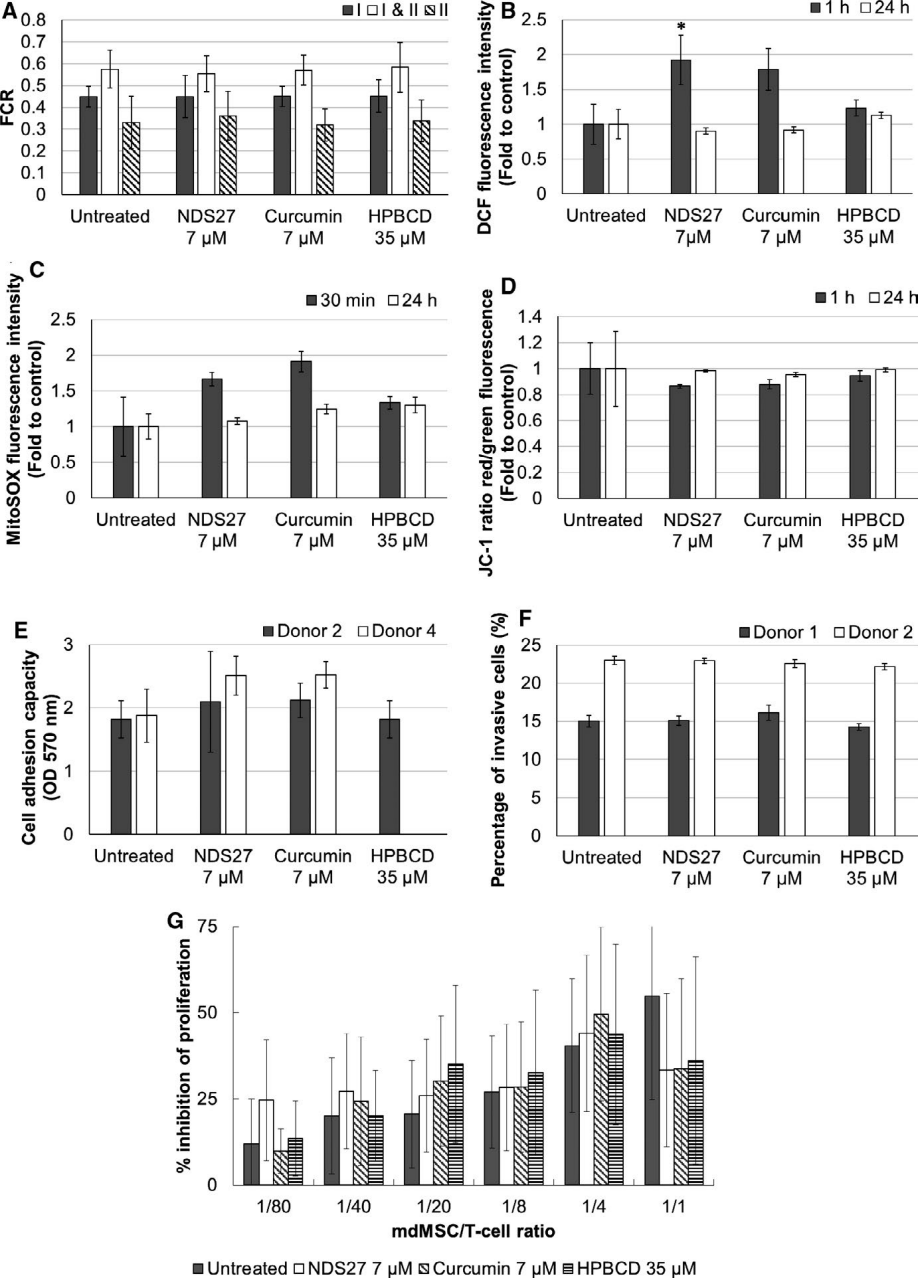
Effects of NDS27 on mitochondrial function and mesenchymal stem cells properties. Cells were treated during 2 h with 7 µmol/L of NDS27 or synthetic curcumin, or with 35 µmol/L of HPβCD. (A) Effect of treatments on respiration complexes (I and II) activity. Data are expressed as Flux Control Ratio ± SD. The experiment was performed on three independent biological replicates. (B) Intracellular ROS and RNS content in mdMSCs one or 24 h after the loading. Data are expressed as mean ± SD of five biological replicates. Statistical comparisons between treated conditions and control 1 h after loading are based on Mann‐Whitney test according to conventional thresholds: *P* <.05 (*). (C) Mitochondrial ROS abundance in mdMSCs 30 min after the loading and 24 h later. Data are expressed as mean ± SD of five biological replicates. (D) Mitochondrial membrane potential in mdMSCs 1 h after the loading and 24 h later. Data are expressed as mean ± SD of five biological replicates. (E) Cell adhesion capacity of mdMSCs 1 h after treatments. Data are expressed as mean of OD at 570 nm ± SD of two independent biological replicates analysed in four technical replicates. (F) Percentage of invasive mdMSCs, 24 h after the treatments. Data are expressed as mean of percentage of invasive cells ± SD of two biological replicates analysed each in five technical replicates. (G) Percentage of inhibition of T cell proliferation ± SD mediated by NDS27‐loaded mdMSCs at different cellular ratios. The experiment was performed on five biological replicates.

Firstly, we showed that the average concentrations of NDS27 and synthetic curcumin required to inhibit by 50% the cell growth (IC_50_) of mdMSCs are both of approximately 50 µmol/L after 24 hours and dropped to 27 µmol/L after 48 and 72 hours of treatment (Figure 1A). mdMSCs appeared thus less sensitive to cytotoxic effects of curcumin than cancerous cell lines (mean IC_50_ of 7.85 µmol/L; NCI database). Moreover, Wang *et al* have demonstrated benefits of a pre‐treatment of rat bone‐marrow MSCs with curcumin (10 µmol/L for 24 hours) on their survival from hypoxia/reoxygenation injury which is a phenomenon that can be encountered during their use for regenerative medicine.^5^ As shown in Appendix S1, continuous exposure to a 7 µmol/L NDS27 treatment provoked limited morphological alterations and only a moderate decreased proliferation of mdMSCs (Figure S1). Because we intend to prime mdMSCs for few hours only and because videomicroscopy analysis showed limited morphological changes of the cells till 24 hours even at high concentration (Figure S1A), we decided to evaluate the internalization of NDS27 in mdMSCs at two working concentrations: a low concentration that appeared non‐toxic, ie 7 µmol/L, and a concentration of 42 µmol/L which is close to the average 24 hours IC_50_. Quantities of curcumin internalized by the cells are concentration‐dependent: approximately 2% of the initial dose put in presence of cells is internalized whatever the duration of exposure duration or the form of curcumin (ie NDS27 or synthetic curcumin) (Figure 1B). Similarly, Moustapha *et al* who quantified the uptake of curcumin in a human liver cell line demonstrated that the internalization of curcumin is indeed already maximal after 5 minutes.^6^ The assessment of the viability and proliferation capacities of pre‐loaded mdMSCs revealed that a loading of 2 hours with 7 µmol/L of NDS27 does not affect these properties (Table S3). Therefore, we selected this priming condition for further investigations. We took advantage of the intrinsic fluorescence of curcumin to observe its subcellular localization under these experimental conditions. A cytosolic punctuate pattern suggested a mitochondrial localization, which was confirmed by observing the partial colocalization of curcumin with MitoTracker, a mitochondria‐specific probe (Figure 1C,D,E). This observation, in concordance with other studies^7^, ^8^ prompted us to assess if NDS27 could modify mitochondrial functions. Indeed, mitochondrial activity could influence the undifferentiated state of MSCs and their differentiation potential.^9^ Although a slight increase in total and mitochondrial ROS levels and a slight drop of the mitochondrial membrane potential were observed just after the loading of mdMSCs with 7 µmol/L of curcumin or NDS27 (Figure 2B,C,D), high resolution respirometry did not evidence any significant impact on mitochondrial respiration and especially the oxygen consumption for complexes I and II (Figure 2A). In addition, the effects observed on ROS production and mitochondrial membrane potential appeared transient as they did not persist 24 hours after the loading (Figure 2B,C,D).

One of the potential clinical applications of NDS27‐loaded mdMSCs could be osteoarthritis that would benefit from the anti‐inflammatory properties of curcumin as well as immunomodulatory and regenerative properties of MSCs. For this purpose, the preservation of mesenchymal properties like their adhesion to cartilage and their immunomodulation potential is essential.^10^, ^11^ We observed herein that adhesion of mdMSCs to fibronectin is very rapid and already complete after 1 hour (Figure S2). Furthermore, the capacity of mdMSCs to adhere to fibronectin was not affected by their loading with NDS27 (Figure 2E). For reparative cell therapy, after their adhesion to cartilage, mdMSCs have to invade the injured cartilage to ensure the structural and functional maintenance.^12^ The evaluation of the invasive capacity of mdMSCs loaded 2 hours with NDS27 at 7 µmol/L showed that the NDS27 loading does not affect the invasiveness of mdMSCs (Figure 2F). Moreover, the expression of mesenchymal surface markers (CD29, CD44, CD73 and CD105) and a pluripotency marker (OCT4) 24, 48 and 72 hours after the loading was also not altered by this loading (Figure S3). Finally, the potential of NDS27‐treated mdMSCs to inhibit T cell proliferation for 24 hours was intact whatever the mdMSCs/T cells ratio (Figure 2G). Importantly, previous studies have nevertheless demonstrated cytoprotective effects of curcumin pre‐treatment (10 µmol/L for 24 hours) on rat bone‐marrow‐ and adipose tissue‐MSCs against oxidative stress induced by H_2_O_2_ exposure thanks to its antioxidant properties.^13^, ^14^ Although the effects of NDS27 observed in the present work are similar to those of synthetic curcumin, NDS27 displays a real advantage as it is water‐soluble compared to curcumin that needs to be dissolved in DMSO.

In conclusion, we found that curcumin loading of mdMSCs is concentration‐dependent and that a short priming of only 2 hours at 7 µmol/L of NDS27 as well as curcumin itself does not alter their mitochondrial function and allows keeping their mesenchymal properties. Altogether, those data illustrate the safety of NSD27 as a curcumin loading agent and allow us to consider the immediate perspectives that include evaluation of the anti‐inflammatory, antioxidant and immunomodulatory properties of NDS27‐loaded mdMSCs in osteoarthritis models.

## CONFLICT OF INTEREST

VM and DS are inventors of NDS27 (WO2009144220A1). DS and JC got patent for mdMSC (WO2015091210). DS is administrator of Bioptis and RevaTis companies (respectively provider of NDS27 and mdMSCs). JC and AN are employees in RevaTis. Other authors have no conflict of interest.

## AUTHOR CONTRIBUTION

**Margaux Colin:** Data curation (equal); Formal analysis (equal); Investigation (equal); Writing‐original draft (equal). **Lola Dechêne:** Data curation (equal); Formal analysis (equal); Investigation (equal); Writing‐original draft (equal). **Justine Ceusters:** Funding acquisition (equal). **Ariane Niesten:** Resources (supporting). **Catherine Demazy:** Resources (supporting). **Laurence Lagneaux:** Methodology (supporting). **Karim Zouaoui Boudjeltia:** Writing‐review & editing (equal). **Thierry Franck:** Project administration (equal); Writing‐review & editing (equal). **Pierre Van Antwerpen:** Writing‐review & editing (equal). **Patricia Renard:** Conceptualization (equal); Supervision (equal); Validation (equal); Writing‐review & editing (equal). **Véronique Mathieu:** Conceptualization (equal); Supervision (equal); Validation (equal); Writing‐review & editing (equal). **Didier Serteyn:** Funding acquisition (equal); Writing‐review & editing (equal).

## Supporting information

App S1

## Data Availability

The data that support the findings of this study are available from the corresponding author upon reasonable request.
