# Supplementary material for: Priming of mesenchymal stem cells with a hydrosoluble form of curcumin allows keeping their mesenchymal properties for cell‐based therapy development
Source: J Cell Mol Med. 2021 Mar 26;25(10):4877–81. doi: 10.1111/jcmm.16403 (PMC8107093; doi:10.1111/jcmm.16403)
Supplement: Supplementary file 1 — App S1 [file JCMM-25-4877-s001.docx]

**Appendix S1**

**Supporting Information**

- 1. **Materials and methods**

All culture materials, reagent and equipment sources are detailed in Key resources tables (section 1.13).

- 1. **Cell lines and compounds**

The equine skeletal mdMSCs from five donors (see section 1.13 Key resources table) were provided by RevaTis (Aye, Belgium). They were cultured in Dulbecco’s modified Eagle’s medium (DMEM) F-12 culture medium supplemented with 20% heat-inactivated fetal bovine serum (HI-FBS), 100 IU/mL of penicillin-streptomycin and 0.5% of amphotericin B at 37°C and 5% CO_2_ according to the recommendations of RevaTis.^1^

NDS27, synthetic curcumin and HPβCD were provided from BiopTis (Aye, Belgium). NDS27 and curcumin were respectively dissolved in water and dimethyl sulfoxide (DMSO) as described by Franck *et al*.^2^

## **Quantitative videomicroscopy**

Computer-assisted phase contrast microscopy was performed as previously described.^3^ Briefly, mdMSCs were seeded in 25 cm^2^ culture flasks and left untreated or treated with NDS27 at 7 µM. Pictures of one field were taken every four minutes during a 72 hours period and further compiled into a short movie. Quantitatively, global growth ratio was determined based on cell counting of pictures corresponding to 12, 24, 36 and 48 hours in comparison to time zero.

## **Quantification of intracellular curcumin by HPLC**

Cells were treated with NDS27 at 7 or 42 µM, or with synthetic curcumin at 42 µM for two, four or six hours and were then washed twice with Dulbecco’s phosphate-buffered saline (DPBS), scrapped in 100 µL of ice-cold methanol and sonicated for 30 seconds. Samples were then centrifuged five minutes at 12,000 g before analysis. The number of cells was determined for each condition to normalize the data of internalization for one million cells. Twenty microliters from the prepared samples were injected into the liquid chromatography system. Analyses were performed with a high-performance liquid chromatography (HPLC) 1100 series. Separation was performed on a Discovery C18 HPLC column (15 cm x 4.6 mm, 5 µm particle size) using 0.2% trifluoroacetic acid acidified water / acetonitrile gradient (Table S1) at a flow rate of 1.0 mL/minute. The detection system was fixed at 425 nm. Data were acquired and integrated using Agilent Chemstation software. The data collected by the chromatographic system were analyzed on the basis of the peak area compared to a standard curve ranging from 1 to 25 µM performed for each experiment with both NDS27 and synthetic curcumin.

| Time (min) | Trifluoroacetic acid (%) | Acetonitrile (%) | Flow rate (mL/min) |
| --- | --- | --- | --- |
| 0 | 70 | 30 | 1 |
| 2.5 | 70 | 30 | 1 |
| 11.5 | 10 | 90 | 1 |
| 12 | 70 | 30 | 1 |
| 14 | 70 | 30 | 1 |

**Table S1.** Sequence of gradient solvent to separate curcumin on Discovery C18 HPLC column.

## **Subcellular localization**

Cells seeded in chambered coverglass Nunc Lab-Tek were treated with 7 μM of NDS27 for 90 minutes 24 hours after the seeding. Then, DMEM-F12 without serum supplementation but containing 7 μM of NDS27 and 100 nM of MitoTracker Red CM-H2XRos replaced the previous medium for 30 minutes to reach two hours of exposure. Cells were washed with DPBS and observed by confocal microscopy (SP5, Leica). Transects were obtained with Fiji software: lines were traced, analyzed with “plot profile” and added to the region of interest (ROI) manager to apply this line on the other fluorescence channel. Quantification of colocalization between NDS27 natural fluorescence and mitochondria probe was performed with Fiji software following the protocol of Dunn *et al*.^4^

- 1. **Viability and proliferation assays**

For each viability assay, cells were seeded in 96 well plates and were grown for 24 hours. To determine the half maximal inhibitory concentration (IC_50_) of the products, cells were treated with NDS27 or synthetic curcumin at concentrations ranging from 10 nM to 100 µM or left untreated for 24, 48 and 72 hours.

To evaluate the capacity of NDS27/curcumin-loaded mdMSCs (i.e. treated) to proliferate, cells were first exposed for two hours to 7 or 42 µM of NDS27 or synthetic curcumin, 35 or 210 µM of HPβCD or left untreated. After this loading treatment, cells were washed once with culture medium and then incubated with culture medium for 24, 48 and 72 additional hours prior to viability evaluation.

### **MTT colorimetric assay**

Cell viability based on a 3-(4,5-dimethylthiazol-2-yl)-2,5-diphenyltetrazolium bromide (MTT) colorimetric assay was performed as described previously.^5^ The viability was estimated using MTT that measured mitochondrial reduction into formazan at 570 nm with a spectrophotometer 680XR (reference wavelength 610 nm).

### **Sulforhodamine B Cell Cytotoxicity assay**

This assay was performed according to the kit instructions. Briefly, at the end of the incubation period, cells were fixed and then stained with Sulforhodamine B (SRB) reagent for 15 minutes prior to the extraction of the colored product. Finally, the absorbance was measured at 570 nm with a microplate spectrophotometer Synergy Mx.

### **Crystal Violet assay**

At the end of the incubation period, cells were fixed with 17.5% formaldehyde for 10 minutes at 4°C prior to their wash with distilled water. Plates were then dried before the addition of 0.1% crystal violet (CV) dissolved in methanol for 15 minutes at room temperature in the dark. After the coloration step, cells were washed with distilled water before the extraction of the colored product with acetic acid solution 33%. The absorbance was measured at 570 nm with a microplate spectrophotometer Synergy Mx.

## **Mitochondrial respiratory capacity**

Cells were treated with NDS27, curcumin or HPβCD for 2 hours and then harvested for mitochondrial-respiratory capacity assay. The substrate-uncoupler-inhibitor titration (SUIT) protocol 1 was applied for the evaluation of mitochondrial respiration as described by Votion *et al*.^6^ Measures were recorded by high-resolution respirometry (Oxygraph-2k chamber, Oroboros instrument). Flux control ratio (FCR) was calculated as previously described.^7^

## **Measurement of total reactive oxygen species (ROS) and reactive nitrogen species (RNS), mitochondrial ROS and evaluation of mitochondrial membrane potential**

For each of the three assays below, seeded cells were treated during two hours with NDS27 (7 µM), synthetic curcumin (7 µM), HPBCD (35 µM), DMSO (0.07%), or left untreated. Cells were then washed once with culture medium prior to the addition of the probes. Analyses were performed either directly after the loading or 24 hours later. For this later condition, cells were washed once with culture medium and then left in culture medium for 24 hours before staining described below. Analyses of the samples were conducted on a total of 10,000 events for each sample with a Beckman Gallios cytometer.

### **Total ROS and RNS measurement**

Intracellular amount of total ROS and RNS has been evaluated using the diacetylated form of 2’,7’-dichlorodihydrofluorescein diacetate (DCFH-DA). ROS and RNS oxidize DFCH into 2’,7’-dichlorofluorescein (DCF) which emits a green fluorescence measured by flow cytometry.^8^ Following the wash of cells, DCFH-DA (20 µM) prepared in DPBS was added for one hour. Cells were then detached, centrifuged and resuspended in DPBS before the fluorescence analysis by flow cytometry.

### **Mitochondrial ROS measurement**

The production of mitochondrial ROS including superoxide anions has been studied using the MitoSOX reagent.^9^ Following the wash of cells, MitoSOX (1 µM) prepared in DPBS was added for 30 minutes. Cells were then detached, centrifuged and resuspended in DPBS before the fluorescence analysis by flow cytometry.

### **Mitochondrial membrane potential**

The evaluation of the integrity of the mitochondrial membrane potential was performed using a cationic fluorescent dye: 1,1′,3,3′-tetraethylbenzamidazolocarbocyanin iodide (JC-1) that concentrates into the mitochondria leading to aggregates that fluoresce in red (Emission: 590 nm). This process is impaired when the mitochondrial membrane potential is altered, and JC-1 remains in monomers that fluoresce in green (Emission: 529 nm).^10^ Directly after their loading or 24 hours later, cells were detached and centrifuged. Then, cells were stained with the JC-1 probe prepared in DPBS at 3.45 µg/mL for 20 minutes. After two washes with DPBS, cells were resuspended in 150 µL of DPBS for fluorescence analyses. The red/green fluorescence ratio indicates the mitochondrial membrane potential. ^10^

## **Cellular adhesion capacity**

Cells pre-treated for two hours with curcumin or NDS27 at 7 µM were seeded in 96 wells plates previously coated with human plasma fibronectin (0.2 mg/mL in DPBS). They were allowed to adhere for one and 21 hours at 37°C and 5% CO_2_. After each time point, cells were washed once with DPBS and fixed with 17.5% formaldehyde for 10 minutes at 4°C prior to two washing steps with DPBS. Plates were dried before crystal violet staining with 0.1% crystal violet dissolved in methanol for 15 minutes at room temperature in the dark. Wells were then washed with distilled water before the extraction of the colored product with acetic acid solution 33%. The absorbance was measured at 570 nm with a microplate spectrophotometer Synergy Mx.

## **Cellular invasion capacity**

Invasion ability of mdMSCs was studied *in vitro* using fibronectin-coated (0.2 mg/mL) Boyden invasion chambers (96 well plates; PromoKine). Cells pre-treated for two hours with curcumin or NDS27 at 7 µM were seeded in top chamber in culture medium supplemented with 0.5% HI-FBS while the bottom chambers contained culture medium supplemented with 10% HI-FBS. After 24 hours incubation, the upper parts of the top chambers were washed with a Q-tip to remove cells that did not migrate. Cells having passed the fibronectin matrix and the polyethylene terephthalate membrane were then dissociated and stained according to manufacturer’s instruction. Finally, the percentage of invasion was determined using a standard curve of seven points from 100,000 to 1,562 cells.

## **1.10 T-cell proliferation assays**

This assay measures the ability of mdMSCs to inhibit the proliferation of purified T-lymphocytes. This immunomodulation property of mdMSCs were evaluated as previously described.^1,11^

**1.11 Real-time quantitative polymerase chain reaction (RT-qPCR)**

Total RNA was extracted using ReliaPrep RNA Miniprep Systems. Two micrograms of total RNA were reverse transcribed using GoScript Reverse Transcription mix Oligo(dT) for cDNA synthesis. GoTaq qPCR Master Mix was then used to perform amplification on a Viia7 equipment. Primers listed in table S2 were used at a concentration of 300 nM. All results were normalized to the mRNA abundance of enolase (i.e. housekeeping gene) using the 2^-∆∆Ct^ method and expressed relative to the untreated cells condition.

**Table S2**. Primers used for RT-qPCR.

| **Primers** | | | |
| --- | --- | --- | --- |
| **Gene** | **Forward** | **Reverse** | **Efficiency (%)** |
| Enolase | GTGCAGCCAACTTCAGTGAA | CCAGCTTTCCCAATGGCATT | 97 |
| CD29 | AGAATGTATACAAGCAGGGCCA | TTATATCTTTGGAGCCTCTGGG | 93 |
| CD44 ^12^ | CCCACGGATCTGAAACAAGTG | TTCTGGAATTTGAGGTCTCCGTAT | 87 |
| CD73 ^12^ | GGGATTGTTGGATACACTTCAAAAG | GCTGCAACGCAGTGATTTCA | 87 |
| CD90 | GGTCCTCTACTTATCCAGCTTCA | CCAGTTTGTCTCGGAGCACA | 95 |
| CD105 ^13^ | GACGCCAATCACAACATACA | TCCACATAGGACGCTACGAC | 85 |
| OCT4 ^14^ | GGTACGAGTGTGGTTCTGC | GTGCCAGGGGAAAGGATACC | 88 |

**1.12 Statistical analysis**

Statistical analyses of subcellular localization were performed by Prism using a Mann-Whitney test. Statistical analyses of mitochondrial respiratory capacity and T-cell proliferation assays were performed by Prism using the unpaired T test. Statistical analyses of global growth ratio, total ROS and RNS, mitochondrial superoxide anion and mitochondrial membrane potential were performed by Statistica using Mann-Whitney test. Statistical analyses of RT-qPCR were performed by Prism using the Kruskal-Wallis test followed by the Dunn’s multiple comparison.

| **mdMSCs donors** | | |
| --- | --- | --- |
| **Number of the donor** | **Sex** | **Age at the time of collection** |
| **1** | ♀ | 22 years |
| **2** | ♀ | 16 years |
| **3** | ♀ | 13 years |
| **4** | ♀ | 12 years |
| **5** | ♀ | 10 years |

**1.13 Key resources tables**

| **Culture medium, culture supplement, chemicals, recombinant proteins and kits** | |
| --- | --- |
| **Reagent or resource** | **Source** |
| DMEM/F-12 | Gibco, Thermofisher, Dilbeek, Belgium |
| Heat-inactivated foetal bovine serum (HI-FBS) |  |
| Penicillin-streptomycin |  |
| Amphotericin B |  |
| DPBS |  |
| Methanol | VWR International, Oud-Heverlee, Belgium |
| Acetonitrile |  |
| Dimethyl sulfoxide (DMSO) |  |
| Trifluoroacetic acid | Sigma-Aldrich, Diegem, Belgium |
| 3-(4,5-dimethylthiazol-2-yl)-2,5-diphenyltetrazolium bromide (MTT) |  |
| Crystal violet (CV) |  |
| 2’,7’-dichlorodihydrofluorescein diacetate (DCFH-DA) |  |
| Human plasma fibronectin |  |
| MitoTracker Red CM-H2XRos | Invitrogen, Thermofisher, Dilbeek, Belgium |
| MitoSOX reagent |  |
| Sulforhodamine B Cell Cytotoxicity assay | Abcam, Cambridge, United-Kingdom |
| 1,1′,3,3′-tetraethylbenzamidazolocarbocyanin iodide (JC-1) | Enzo Life Sciences, Brussels, Belgium |
| Primers | IDT, Leuven, Belgium |
| Cell invasion assay kit, fibronectin, 8 µm, 96 well | PromoKine, Bio-connect, The Netherlands |
| ReliaPrep RNA Miniprep Systems | Promega Benelux, Leiden, The Netherlands |
| GoScript Reverse Transcription mix Oligo(dT) |  |
| GoTaq qPCR Master Mix |  |

| **Culture materials** | |
| --- | --- |
| **Resource** | **Source** |
| 25 cm² culture flasks | Sarstedt AG & CO, Nümbrecht, Germany |
| 96 well plates |  |
| Coverglass Nunc Lab-Tek | Thermofisher, Dilbeek, Belgium |
| **Equipment** | |
| **Resource** | **Source** |
| HPLC 1100 series | Agilent Technologies, Santa Clara, USA |
| Diode Array Detector G1315B |  |
| Discovery C18 HPLC column (15 cm x 4,6 mm, 5 µm particle size) | Supelco Analytical, Sigma-Aldrich, Diegem, Belgium |
| confocal microscopy (TCS SP5 II) | Leica Microsystems, Wetzlar, Germany |
| Spectrophotometer 680XR | Bio-Rad Laboratories, Berkeley, CA, USA |
| Spectrophotometer Synergy Mx | BioTek, France |
| Oxygraph-2k chamber, Oroboros instrument | Oroboros instruments, Innsbruck, Austria |
| Flow cytometer | Gallios, Beckmann Coulter, Analis, Suarlee, Belgium |
| Odyssey Infrared Imager | Li-Cor Biosciences, Lincoln, NE, USA |
| Viia7 equipment | Applied Biosystems, Thermo Fisher Scientific Leusden, The Netherlands |
| **Software** | |
| **Resource** | **Source** |
| Agilent Chemstation software | Agilent Technologies, Santa Clara, USA |
| Fiji software | National Institutes of Health, MD, USA |
| Odyssey V3.O software | Li-Cor Biosciences, Lincoln, NE, USA |
| Statistica | Statsoft, Tulsa, OK, USA |
| Prism | GraphPad Software, San Diego, CA, USA |

- 1. **References**

1. Ceusters J, Lejeune JP, Sandersen C, Niesten A, Lagneaux L, Serteyn D. From skeletal muscle to stem cells: An innovative and minimally-invasive process for multiple species. *Sci Rep*. 2017. doi:10.1038/s41598-017-00803-7

2. Franck T, Aldib I, Zouaoui Boudjeltia K, et al. The soluble curcumin derivative NDS27 inhibits superoxide anion production by neutrophils and acts as substrate and reversible inhibitor of myeloperoxidase. *Chem Biol Interact*. 2019;297:34-43. doi:10.1016/j.cbi.2018.10.008

3. Debeir O, Mégalizzi V, Warzée N, Kiss R, Decaestecker C. Videomicroscopic extraction of specific information on cell proliferation and migration in vitro. *Exp Cell Res*. 2008;314(16):2985-2998. doi:10.1016/j.yexcr.2008.06.010

4. Dunn KW, Kamocka MM, McDonald JH. A practical guide to evaluating colocalization in biological microscopy. *AJP Cell Physiol*. 2011;300(4):C723-C742. doi:10.1152/ajpcell.00462.2010

5. Mosmann T. Rapid colorimetric assay for cellular growth and survival: Application to proliferation and cytotoxicity assays. *J Immunol Methods*. 1983;65(1-2):55-63. doi:10.1016/0022-1759(83)90303-4

6. Votion DM, Gnaiger E, Lemieux H, Mouithys-Mickalad A, Serteyn D. Physical fitness and mitochondrial respiratory capacity in horse skeletal muscle. *PLoS One*. 2012;7(4). doi:10.1371/journal.pone.0034890

7. Pesta D, Gnaiger E. High-resolution respirometry: OXPHOS protocols for human cells and permeabilized fibers from small biopsies of human muscle. *Methods Mol Biol*. 2012;810:25-58. doi:10.1007/978-1-61779-382-0_3

8. Eruslanov E, Kusmartsev S. Identification of ROS using oxidized DCFDA and flow-cytometry. *Methods Mol Biol*. 2010;594:57-72. doi:10.1007/978-1-60761-411-1_4

9. Kauffman M, Kauffman M, Traore K, et al. MitoSOX-Based Flow Cytometry for Detecting Mitochondrial ROS. *React Oxyg Species*. 2016;2(5). doi:10.20455/ros.2016.865

10. Elefantova K, Lakatos B, Kubickova J, Sulova Z, Breier A. Detection of the mitochondrial membrane potential by the cationic dye JC-1 in l1210 cells with massive overexpression of the plasma membrane ABCB1 drug transporter. *Int J Mol Sci*. 2018;19(7). doi:10.3390/ijms19071985

11. Fayyad-Kazan H, Faour WH, Badran B, Lagneaux L, Najar M. The immunomodulatory properties of human bone marrow-derived mesenchymal stromal cells are defined according to multiple immunobiological criteria. *Inflamm Res*. 2016;65(6):501-510. doi:10.1007/s00011-016-0933-2

12. Barrachina L, Remacha AR, Romero A, et al. Effect of inflammatory environment on equine bone marrow derived mesenchymal stem cells immunogenicity and immunomodulatory properties. *Vet Immunol Immunopathol*. 2016. doi:10.1016/j.vetimm.2016.02.007

13. Zahedi M, Parham A, Dehghani H, Mehrjerdi HK. Stemness signature of equine marrow-derived mesenchymal stem cells. *Int J Stem Cells*. 2017. doi:10.15283/ijsc16036

14. Sharma R, Livesey MR, Wyllie DJA, et al. Generation of functional neurons from feeder-free, keratinocyte-derived equine induced pluripotent stem cells. *Stem Cells Dev*. 2014;23(13):1524-1534. doi:10.1089/scd.2013.0565

1. **Supplementary results**

**
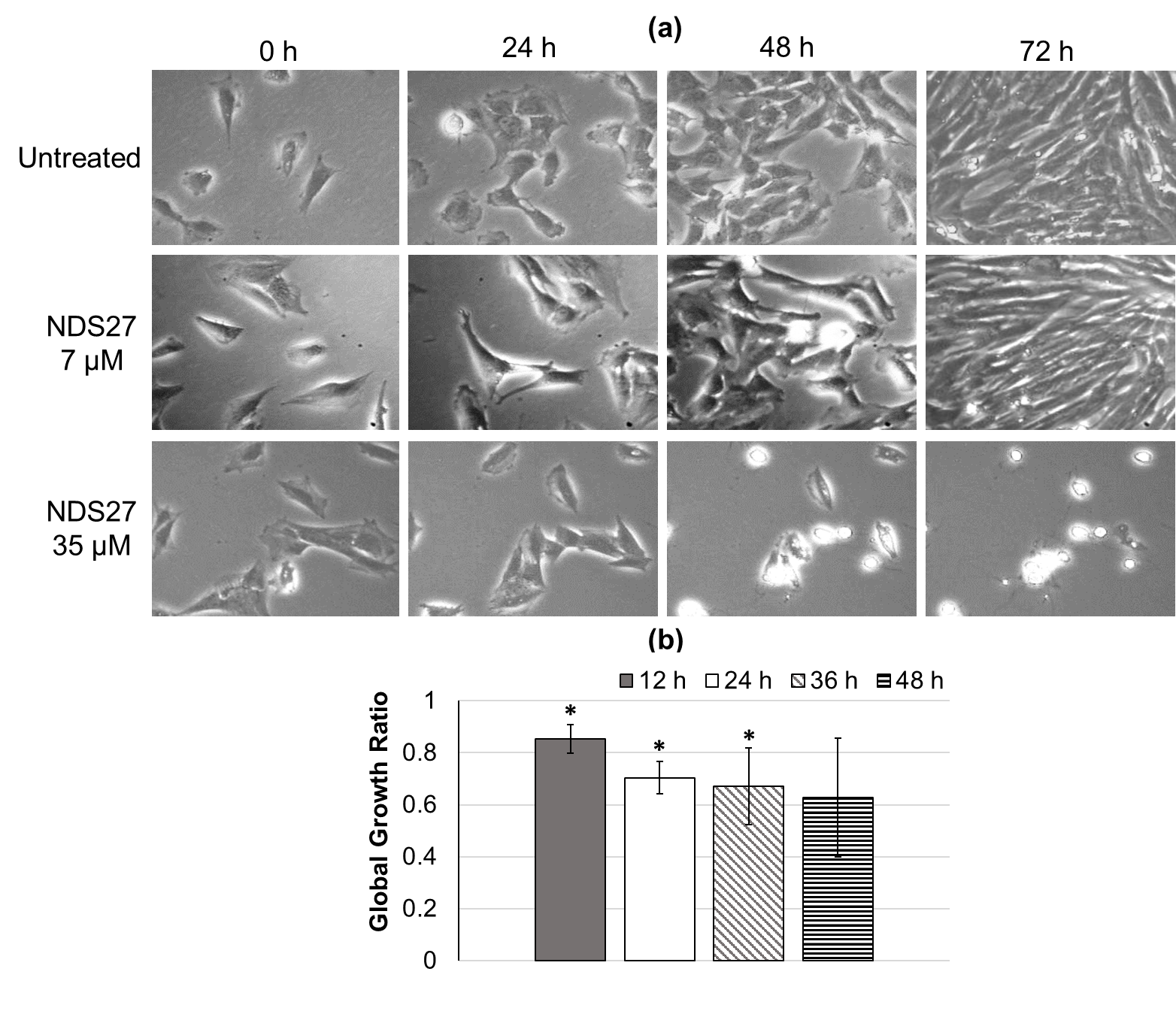
**

**Figure S1**. **(a)** Illustration of the in vitro effects of NDS27 treatments at 7 and 35 µM on equine mdMSCs of donor 3, obtained by videomicroscopy. Figures are representative of one experiment performed in three replicates. **(b)** Global growth ratio, calculated from videomicroscopy analyses illustrated in (a) in equine mdMSCs cells after 12, 24, 36 and 48 hours treatments with NDS27 at 7 µM. Results are expressed as the mean growth ratio between treated cells relative to untreated control arbitrarily set to 1 ± SD of three independent biological replicates. Statistical comparison between untreated cells and treated cells for each duration of treatment is based on Mann-Whitney test according to conventional thresholds: p < 0.05 (*).

|  | **24h** | | | **48h** | | | **72h** | | |
| --- | --- | --- | --- | --- | --- | --- | --- | --- | --- |
|  | **MTT** | **CV** | **SRB** | **MTT** | **CV** | **SRB** | **MTT** | **CV** | **SRB** |
| **HPBCD**  **35 µM** | 100 ± 3 | 100 ± 10 | 100 ± 19 | 99 ± 6 | 97 ± 7 | 104 ± 7 | 99 ± 5 | 103 ± 6 | 93 ± 10 |
| **curcumin 7 µM** | 105 ± 5 | 95 ± 9 | 110 ± 36 | 112 ± 9 | 93 ± 12 | 118 ± 13 | 110 ± 7 | 92 ± 18 | 104 ± 16 |
| **NDS27 7 µM** | 102 ± 5 | 93 ± 10 | 119 ± 25 | 103 ± 3 | 95 ± 7 | 115 ± 9 | 104 ± 7 | 104 ± 6 | 98 ± 7 |
| **HPBCD 210 µM** | 98 ± 2 | 106 ± 23 | 106 ± 20 | 99 ± 5 | 97 ± 11 | 107 ± 12 | 101 ± 9 | 99 ± 2 | 95 ± 9 |
| **curcumin 42 µM** | 102 ± 8 | 81 ± 6 | 102 ± 18 | 91 ± 13 | 67 ± 17 | 108 ± 10 | 101 ± 9 | 71 ± 21 | 87 ± 13 |
| **NDS27**  **42 µM** | 93 ± 8 | 74 ± 9 | 81 ± 18 | 81 ± 9 | 66 ± 8 | 83 ± 10 | 86 ± 12 | 72 ± 11 | 76 ± 14 |

**Table S3**. Percentage of viable equine mdMSCs, obtained by MTT, crystal violet (CV) and SRB assay, 24 hours after the loading with NDS27 or synthetic curcumin at 7 or 42 µM, or with HPβCD at 35 or 210 µM. Data are expressed as the mean of viable cells relative to untreated cells (100 %) ± SD of five biological replicates. Grey boxes highlight a decrease in cell growth of more than 20 %.


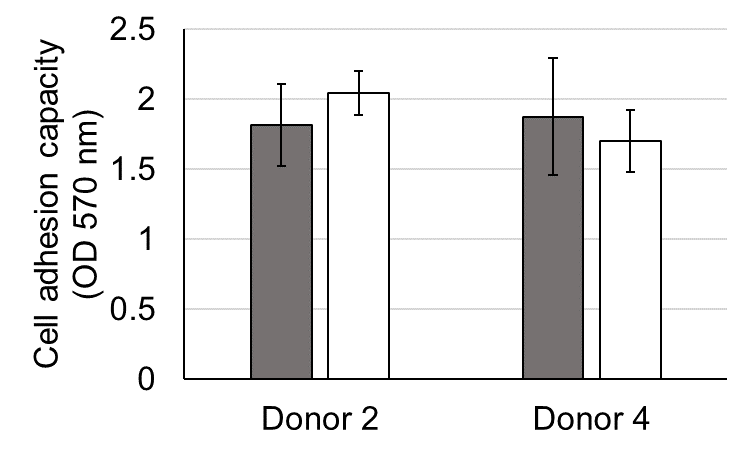


**Figure S2**. Cell adhesion capacity of untreated mdMSCs of donor 2 and 4, one and 21 hours after the seeding ± SD of four technical replicates.


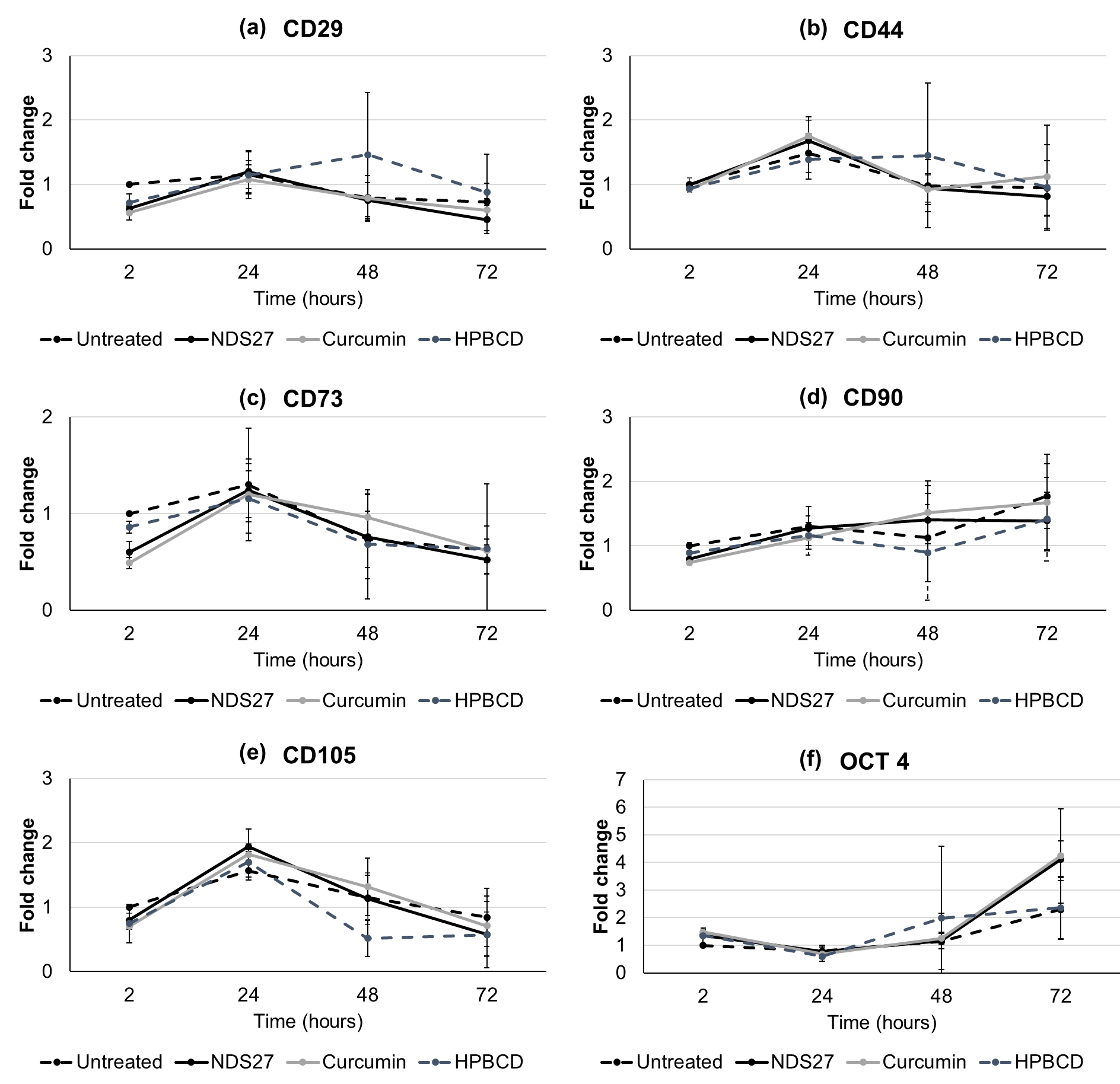


**Figure S3.** Effects of a two hours loading with NDS27 (7 µM), synthetic curcumin (7 µM) or HPβCD (35 µM) on the expression of MSCs characteristic genes ((a) CD29, (b) CD44, (c) CD73, (d) CD90, (e) CD105 and (f) OCT4) measured by qPCR. Data are expressed in fold change to housekeeping gene (Enolase) ± SD of three independent biological replicates.
